# Synthetic Inhibitors of Snake Venom Enzymes: Thioesters Derived from 2-Sulfenyl Ethylacetate

**DOI:** 10.3390/ph12020080

**Published:** 2019-05-23

**Authors:** Isabel C. Henao Castañeda, Jaime A. Pereañez, Lina M. Preciado

**Affiliations:** 1 Grupo de Investigación en Productos Naturales Marinos, Departamento de Farmacia, Facultad de Ciencias Farmacéuticas y Alimentarias, Universidad de Antioquia UdeA, Calle 70 No. 52-21, Medellín 050010, Colombia; 2 Programa de Ofidismo/Escorpionismo, Departamento de Farmacia, Facultad de Ciencias Farmacéuticas y Alimentarias, Universidad de Antioquia UdeA, Calle 70 No. 52-21, Medellín 050010, Colombia; andrespj20@gmail.com (J.A.P.); linampr@gmail.com (L.M.P.)

**Keywords:** snakebite, thioester, phospholipase A_2_, snake venom metalloproteinase, molecular docking

## Abstract

Snakebite envenomings are a global public health issue. The therapy based on the administration of animal-derived antivenoms has limited efficacy against the venom-induced local tissue damage, which often leads to permanent disability. Therefore, there is a need to find inhibitors against toxins responsible for local damage. This work aimed to synthesize thioesters derived from 2-sulfenyl ethylacetate and to evaluate the inhibitory effects on two snake venom toxins. Ethyl 2-((4-chlorobenzoyl)thio)acetate (I), Ethyl 2-((3-nitrobenzoyl)thio)acetate (II) and Ethyl 2-((4-nitrobenzoyl)thio)acetate (III) were synthesized and spectroscopically characterized. Computational calculations were performed to support the study. The inhibitory capacity of compounds (I–III) was evaluated on a phospholipase A_2_ (Cdcum6) isolated from the venom of the Colombian rattlesnake *Crotalus durissus cumanensis* and the P-I type metalloproteinase Batx-I isolated from *Bothrops atrox*. I–III inhibited PLA_2_ with IC_50_ values of 193.2, 305.4 and 132.7 µM, respectively. Otherwise, compounds II and III inhibited the proteolytic activity of Batx-I with IC_50_ of 2774 and 1879 µM. Molecular docking studies show that inhibition of PLA_2_ may be due to interactions of the studied compounds with amino acids in the catalytic site and the cofactor Ca^2+^. Probably, a blockage of the hydrophobic channel and some amino acids of the interfacial binding surface of PLA_2_ may occur.

## 1. Introduction

The World Health Organization (WHO) recognized snakebites as one of the most important Neglected Tropical Diseases in 2017. Nearly 5.4 million snakebites occur each year, and more than 95% of cases occur in tropical or developing countries, mostly in rural areas [1]. There are between 81,410 and 137,880 deaths per year and around three times as many cases of permanent disabilities including amputations [2]. A total of 4978 ophidian accidents were reported in Colombia in 2017 [3]. 

Pathophysiological effects observed in snakebite envenomations combine the action of several enzymes, proteins and peptides, such as phospholipases A_2_ (PLA_2_), hemorrhagic metalloproteinases, and other proteolytic enzymes, coagulant components, neurotoxins, cytotoxins and cardiotoxins, among others [4]. Snake venom metalloproteinases (SVMPs) have been shown to participate in the hemorrhagic process by proteolytic degradation of endothelial cell surface proteins and extracellular matrix components, involved in the maintenance of capillary structure and integrity, leading to capillary network disruption, edema and hemorrhage [5]. Phospholipases A_2_ (PLA_2_) are calcium-depending enzymes that hydrolyze the sn2 ester bond of glycerophospholipids, inducing systemic and local myotoxicity, myonecrosis, neurotoxicity hemolytic activity, among others [6,7].

Nowadays, the only available and accepted therapy for the treatment of snakebites is the intravenous administration of equine or ovine antivenoms [8]. Nevertheless, several reports demonstrated the limited efficacy of antivenom therapy against the local tissue damage caused by venoms [9]. Therefore, there is a need for specific inhibitors of enzymes responsible for local damage to complement conventional parenteral antivenom therapy. 

As usual in drug discovery, inhibitors could be found in natural sources or obtained by chemical synthesis, and in some cases in combination with in silico methodologies. This strategy was used for synthesizing naringenin derivatives which have structural similarity with quercetin, a flavonoid that binds to the active site of a PLA_2_ [10]. 

There are reports of natural and synthetic inhibitors of PLA_2_ from snake venoms including substituted thiobenzoic acid S-benzyl esters [11], Aiplai from leaves of *Azadirachta indica* [12] and cholic and ursodeoxycholic acids [13]. Some of the inhibitors have reported IC_50_ with the same methodology that we use; Pinostrobin, a flavonone isolated from *Renealmia alpinia*, with an IC_50_ of 1.85 mM [14]; and Morelloflavone, a biflavonoid from *Garcinia madruno*, with IC_50_ of 0.38 mM [15].

**Figure 1:**
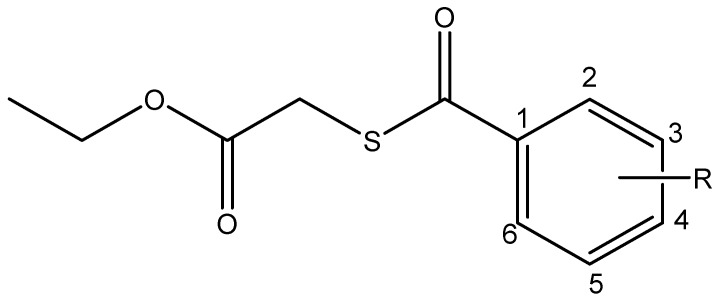
General structure of studied compounds: Ethyl 2-((4-chlorobenzoyl)thio)acetate (I), Ethyl 2-((3-nitrobenzoyl)thio)acetate (II) and Ethyl 2-((4-nitrobenzoyl)thio)acetate (III).

In a previous study we found that substituted thiobenzoic acid S-benzyl esters are inhibitors of an Asp49-PLA_2_ enzyme isolated from the venom of the Colombian *Crotalus durissus cumanensis* rattlesnake, but they have solubility drawbacks [11]. Looking for increasing the polarity and maintaining the biological activity, thioesters derived from 2-sulfenyl ethyl acetate were synthesized changing a phenyl group for an ethyl ester (Figure 1, Table 1). The present study aims to evaluate the inhibitory capacity of these compounds on the PLA_2_ from *C. d. cumanensis* rattlesnake and a PI type SVMP from *Bothrops atrox* venom. 

## 2. Results

### 2.1. Synthesis and Spectroscopical Characterization

We report the synthesis and characterization by GC-MS, IR and NMR of thioesters derived from 2-sulfenyl ethylacetate. Spectra are available as Supplementary Material.

#### 2.1.1. Ethyl 2-((4-chlorobenzoyl)thio)acetate (I)

Colorless crystals; m.p. 55.6 °C; 97% yield; FT-IR (KBr) ν 1734 and 1662 cm^−1^ (C=O); ^1^H NMR (CDCl_3_, 250 MHz) δ (ppm) 7.90 (2H, dt, J = 8, 2 and <1 Hz, H2 and H6); 7.43 (2H, dt, J = 8, 2 y < 1 Hz, H3 and H5); 4.22 (2H, q, J = 7 Hz, O-CH_2_); 3.87 (2H, s, S-CH_2_); 1.29 (3H, t, J = 7 Hz, CH_3_); ^13^C NMR (CDCl_3_, 62.98 MHz) δ (ppm) 189.0 (S-C=O); 168.6 (O-C=O); 140.2 (C4); 134.5 (C1); 129.1 (C2 and C6); 128.8 (C3 and C5); 62.0 (O-CH_2_); 31.5 (S-CH_2_); 14.1 (CH_3_); MS m/z 258 (2%) [M]+; 139 (100%) [C_7_H_4_^35^ClO]+.

#### 2.1.2. Ethyl 2-((3-nitrobenzoyl)thio)acetate (II)

Colorless crystals; m.p. 44.5 °C; 97% yield; FT-IR (KBr) ν 1738 and 1670 cm^−1^ (C=O); ^1^H NMR (CDCl_3_, 250 MHz) δ (ppm) 8.80 (1H, s, H2); 8.46 (1H, br.d, J = 8 Hz, H4); 8.30 (1H, br.d, J = 8 Hz, H6); 7.70 (1H, t, J = 8 Hz, H5); 4.26 (2H, q, J = 7 Hz, O-CH_2_); 3.94 (2H, s, S-CH_2_); 1.32 (3H, t, J = 7 Hz, CH_3_); ^13^C NMR (CDCl_3_, 62.98 MHz) δ (ppm) 188.4 (S-C=O); 168.2 (O-C=O); 147.3 (C3); 138.4 (C1); 132.9 (C6); 130.0 (C5); 129.7 (C4); 122.4 (C2); 62.2 (O-CH_2_); 31.7 (S-CH_2_); 14.1 (CH_3_); MS m/z 269 (2%) [M]+; 150 (100%) [C_7_H_4_O_3_N]+.

#### 2.1.3. Ethyl 2-((4-nitrobenzoyl)thio)acetate (III)

Colorless crystals; m.p. 56.2 °C; 97% yield; FT-IR (KBr) ν 1730 and 1668 cm^−1^ (C=O); 1H NMR (CDCl_3_, 250 MHz) δ (ppm) 8.34 (2H, dt, J = 8, 2 y < 1 Hz, H3 and H5); 8.15 (2H, dt, J = 8, 2 y <1 Hz, H2 and H6); 4.27 (2H, q, J = 7 Hz, O-CH_2_); 3.95 (2H, s, S-CH_2_); 1.33 (3H, t, J =7 Hz, CH_3_); ^13^C NMR (CDCl_3_, 62.98 MHz) δ (ppm) 188.7 (S-C=O); 168.0 (O-C=O); 150.7 (C4); 140.6 (C1); 128.4 (C2 and C6); 123.9 (C3 and C5); 59.9 (O-CH_2_); 31.7 (S-CH_2_); 14.0 (CH_3_); MS m/z 269 (2%) [M]+; 150 (100%) [C_7_H_4_O_3_N]+.

### 2.2. Biological Activity

#### 2.2.1. Inhibition of PLA_2_ activity

**Figure 2:**
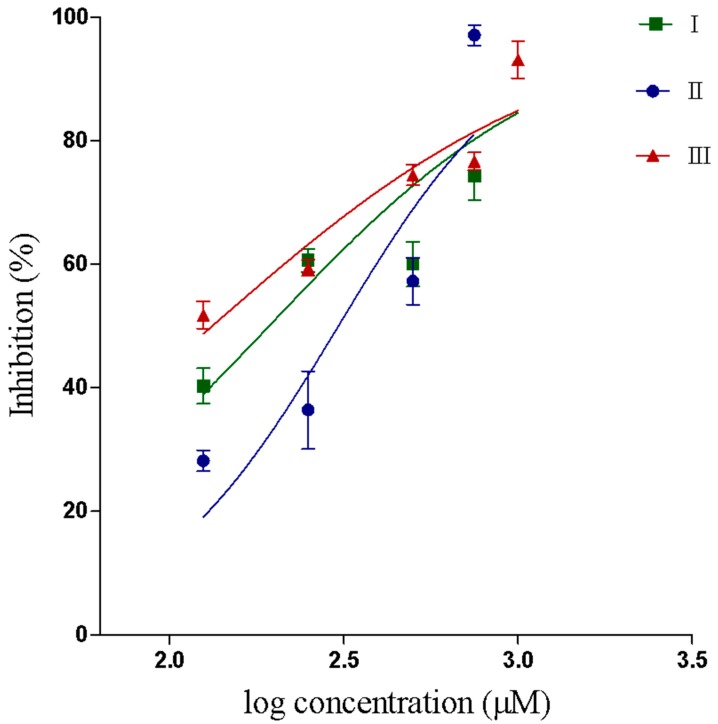
IC_50_ curve showing inhibition of the PLA_2_ enzymatic activity by compounds I, II and III.

The synthetized compounds inhibited the PLA_2_ activity in a dose dependent way. IC_50_ values were 193.2, 305.4 and 132.7 μM for compounds I, II and III respectively. The results and confidence interval values are shown in Table 2. The determination of IC_50_ value was determined from a logistic-dose response curve (Figure 2).

#### 2.2.2. Inhibition of Proteolytic Activity

**Figure 3:**
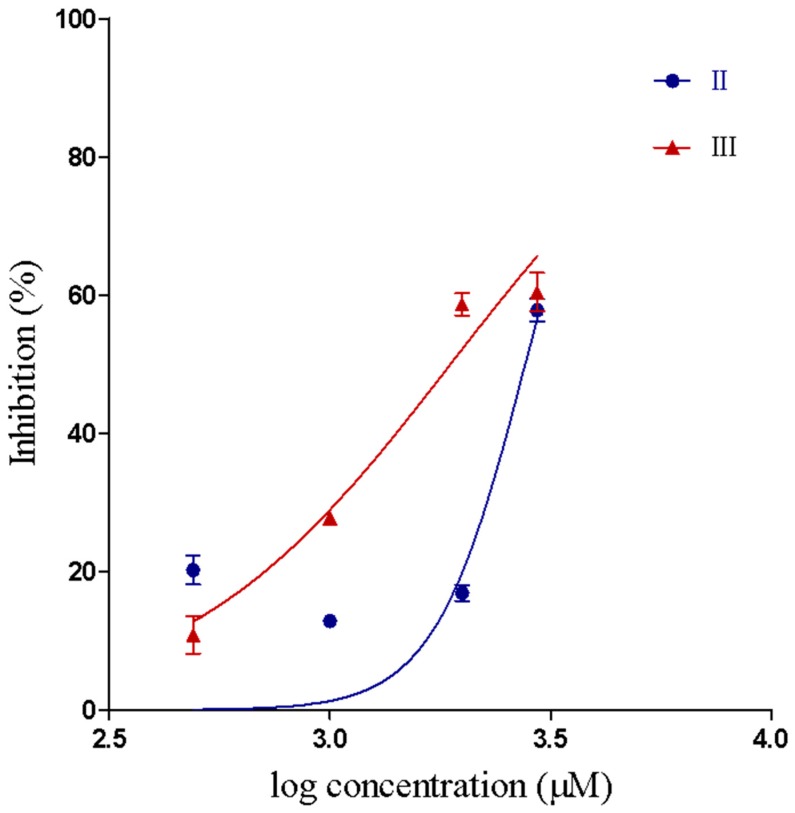
IC_50_ curve showing inhibition of the metalloproteinase proteolytic activity by compounds II and III.

Compounds II and III inhibited the proteolytic activity of Baxt-I on gelatin, in a concentration-dependent manner (Figure 3). Proteolytic IC_50_ and confidence intervals values are shown in Table 2. The concentrations of compounds I–III used in this assay did not induce proteolytic activity on gelatin. However, for compound I the proteolytic IC_50_ could not be determined because 70% of Batx-I proteolytic activity was still observed even at a concentration of 2000 µM.

### 2.3. Computational Studies

#### 2.3.1. Quantum Chemical Calculations

**Figure 4:**
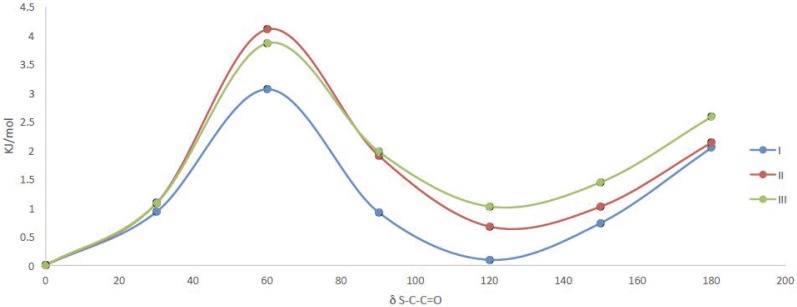
Potential energy curve around the dihedral angle δ S-C-C=O for compounds I, II and III at B3LYP/6-31++G(d,p) level of approximation.

The bridge that connect thioester and ester moieties was explored through a potential energy curve around the dihedral angle δ S-C-C=O using a B3LYP/6-31++G(d,p) approximation. We found two minima at 0° and at 120° with a small energy difference (1 kJ/mol) that indicate the coexistence of both conformers (Figure 4). 

The geometric parameters and vibrational frequencies for both conformers were calculated at the same level of theory. The dihedral angle values calculated for the thioester and ester moieties are presented in Table 3. 

#### 2.3.2. Molecular Docking

To suggest the mechanism of inhibition of the PLA_2_, docking studies with the active compounds were performed using the available protein structure (PDB code 2QOG).

Docking conformations with the lowest binding energy were selected and described. The observed binding free energies with the enzyme PLA_2_ were −23.0; −24.7 and −23.9 kJ/mol for compounds I, II and III, respectively. 

**Figure 5:**
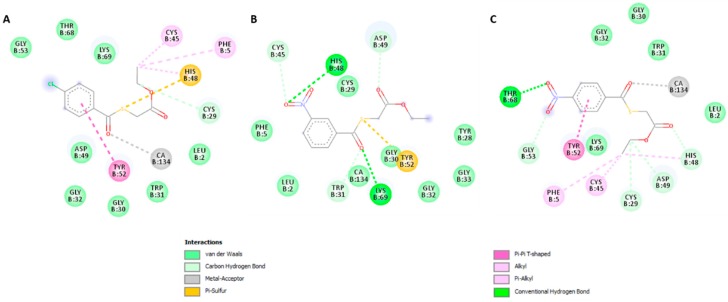
Docking results PLA_2_ with compounds I (**A**), II (**B**) and III (**C**).

**Figure 6:**
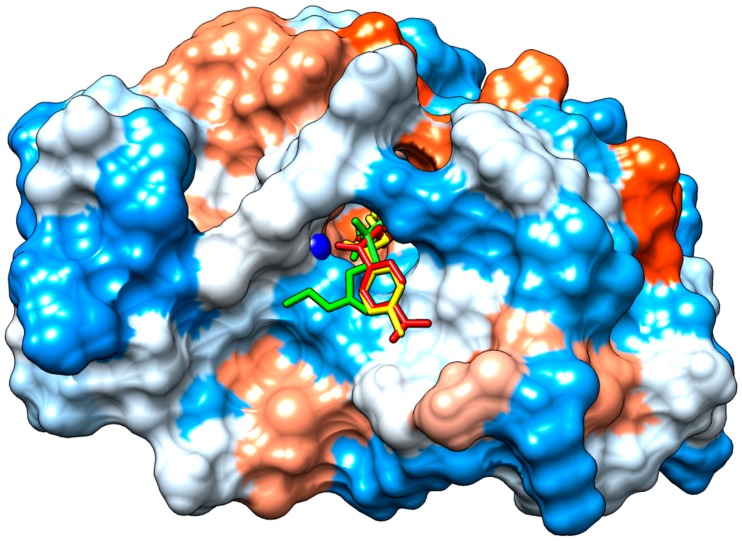
Binding of compounds I, II and III to the substrate binding cleft of the PLA_2_. The red areas of the surface represent the acid regions; the white areas represent the neutral and the blue areas the basic regions. The blue sphere represents Ca^2+^. Compound I (yellow), II (green) and III (red).

Docking results suggested that compounds I–III could form interactions with the residues His 48, Asp 49 and the cofactor Ca^2^+, belonging to the PLA_2_’s active site. In addition, these compounds may interact with amino acids located at the enzyme’s hydrophobic channel and interfacial binding surface, blocking the free access of glycerophospholipids to the active site of the enzyme (Figure 5 and Figure 6). 

## 3. Discussion

Several PLA_2s_ and SVMPs inhibitors with IC_50_ values in the nano and micromolar range for the neutralization of different activities of these toxins have been reported [11,12,13,14]. However, some inhibitors have poor water solubility and low specificity that make their clinical applications difficult. Thus, the aim of this work was to synthesize thioester compounds with improved water solubility with respect to substituted thiobenzoic acid S-benzyl esters reported in a previous work [11], while maintaining the expected biological activity as PLA_2_ inhibitors. The condensation reaction is a high-yielding synthesis (97%) and the strategy was to utilize the same benzoyl chlorides used in the previous study, with a different thiol. The new selected reactant gives us the possibility to obtain the thioester moiety, and also an additional ester moiety in its structure. This change allows the increase in the number of hydrogen acceptors, improving the solubility (lower values of calculated partition coefficient Log *P*). The calculated Log *P* for the previous reported thiobenzoic acid S-benzyl esters were between 3.84 and 4.58, and for compounds I–II were between 2.48 and 3.23.

Compounds I–III have both thioester and ester moiety in their structure. Thioester (X=S) and ester (X=O) moieties usually present a synperiplanar configuration around the δO=C-X-C dihedral angle as the more stable conformer. The results obtained for compounds I, II and III are in agreement with previous reports [11,16,17].

Envenomations induced by viperid snakebites are characterized by local and systemic bleeding. Local effects are associated with a pronounced local tissue damage, while hemodynamic alterations predominate in the systemic effects [18,19]. Both enzymes studied in this work, PLA_2_s and SVMPs contribute to this pathogenesis inducing hemorrhage, myonecrosis, dermonecrosis, blister formation and edema [5,7,20]. The described effects are difficult to neutralize by antibodies due to their rapid symptoms after envenomation [21]. Therefore, it is important to find SVMPs and PLA_2_s inhibitors, like synthetic compounds I–III, that can be administered at the bite site. 

The enzymatic activity of a PLA_2_ is determined by three principal factors: the integrity of the active site (residues His48, Asp49, Tyr52, Asp99), coordination of Ca^2+^ cofactor (residues Tyr28, Gly30, Gly32 and Asp49) and the adsorption of the enzyme onto the lipid–water interface of the phospholipids membrane bilayer (interfacial binding surface) [22], hence they are crucial to study the inhibition mechanism. The molecular docking study suggests that both stable conformations of the studied compounds may interact by either van der Waals or H-bond with amino acids His48 and Asp49 blocking catalytic cycle of PLA_2_. The catalytic mechanism implies water activation by His48 for the subsequent nucleophilic attack of sn2 ligation of the glycerophospholipids that will be hydrolyzed, and carboxylate of the Asp49 side chain stabilizes the oxyanion formed after the nucleophilic attack [22]. Catalytic activity and substrate binding of snake venom phospholipases need submicromolar concentrations of calcium ions. Recently, molecular dynamic studies have demonstrated that calcium induces atomistic movements and conformational changes in snake venom PLA_2_ which led to the formation of a widened cleft at the active site of calcium bound PLA_2_ when compared with free PLA_2_ [23]. This ion is also available for binding the substrate phosphate group [22]. Molecular docking also suggested van der Waals interactions between compounds I, II and III with Gly30 and coordination with Ca^2+^ that could destabilize metal coordination and block enzymatic catalysis. 

The interfacial binding surface of the PLA_2_s mediates the adsorption of the enzyme onto the lipid–water interface of the phospholipid membrane bilayer. Residues Trp31 and Lys69 are part of this structure. Our molecular docking results suggested that all compounds may form van der Waals or π-alkyl interactions with the mentioned residues, thus, the binding of the substrate to the PLA_2_ active site may be blocked. Similar findings were reported in substituted thiobenzoic acid S-benzyl esters at 50 μM and obtained inhibition percentages higher than 50% for three of the four assayed compounds [11]. 

There is a type of snake venom PLA_2_s catalytically inactive (PLA_2_-like myotoxins) due to lack of Ca^2+^ coordination by a natural mutation in which the residue aspartate at position 49 is substituted with a lysine generating the loss of the catalytic activity. However, these toxins are able to induce local mionecrosis by a mechanism dependent of two different sites forming a putative membrane-docking site (MDoS) and a putative membrane disruption site (MDiS) [24]. Different small molecules inhibitors have been described by their inhibition against PLA2-like myotoxins through the binding at just one critical region related to the myotoxic mechanism: the MDoS, MDiS or hydrophobic channel. Recently was demonstrated that chicoric acid binds at the entrance of the hydrophobic channel and clusters of a PLA_2_-like myotoxin that participate in membrane disruption [25]. We hypothesized that thioester compounds could inhibit PLA_2_-like myotoxic activity interacting with amino acids located at the hydrophobic channel, since some amino acids of this structure (Leu2 and Phe5) are involved in the interaction with the Asp49-PLA_2_ tested in this study (Figure 5). Nevertheless, this must be confirmed in future studies. 

Compounds I–III have IC_50_s between 132.7 and 305.4 μM against PLA_2_ enzymatic activity (*p* > 0.05) and are much more active than inhibitors like Pinostrobin and Moreloflavone with IC_50_s determined by the same method of 1.85 and 0.38 mM, respectively [14,15]. Compounds I and III have an electronegative substitution in the para position of the aromatic ring and comparable IC_50_s, whereas compound II is substituted in the meta position. Besides these subtle differences, our docking results suggested that compounds I and III may interact in a similar position with the benzoyl ring at the beginning of the hydrophobic channel and thioester moiety at the middle of the hydrophobic pocket, near to the active site of the enzyme. Instead, compound II was located in an opposite way with phenyl ring near to the active site of the PLA_2_, which may explain the difference in its inhibition capacity. In order to improve the inhibitory potency of compounds, some structural modifications, such as the substitution with a metal binding group can be done with the aim to improve their interaction with catalytically active PLA_2_ Asp49. 

Synthesized compounds were also tested to inhibit the enzymatic activity of a SVMP, however, only II and III showed some ability to inhibit the enzyme with IC_50_ values about 10 times higher than those found for PLA_2_. This talk about their specificity for PLA_2_s, however these compounds may give the possibility to partially also inhibit SVMPs present in snake venoms. Nevertheless, this hypothesis should be addressed in future studies with whole venom and in vivo assays. 

## 4. Materials and Methods

### 4.1. General

Reagents were purchased from Merck, Sigma-Aldrich and Acros organics with commercial, analytic or HPLC grade.

Solvents were evaporated from solutions in a rotary evaporator Heidolph Laborota 4010 equipped with a ROTAVAP valve control. 

### 4.2. Syntheses

Ethyl 2-((4-chlorobenzoyl)thio)acetate (I), Ethyl 2-((3-nitrobenzoyl)thio)acetate (II) and Ethyl 2-((4-nitrobenzoyl)thio)acetate (III) (Figure 1) were prepared with Ethyl 2-mercaptoacetate (1 mmol) dissolved in pyridine and the corresponding substituted-benzoyl chloride (1 mmol) was added to this dissolution. (4-chlorobenzoyl chloride, 3-nitrobenzoyl chloride and of 4-nitrobenzoyl chloride for I, II and III respectively). The reaction mixture was stirred for 1 h at room temperature. The reaction mixture was treated with 1 M hydrochloric acid (5 mL) and methylene chloride (5 mL) and washed thoroughly with distilled water. The organic layer was dried over sodium sulfate anhydride, the solvent was removed in a rotatory evaporator and the residue was recrystallized from methanol and dried under vacuum. 

### 4.3. Spectroscopical Characterization

Melting points (m.p.) were recorded in an Electrothermal 9100 apparatus. Infrared spectra in KBr pellets were measured between 4000 and 400 cm^−1^ (4 cm^−1^ resolution) with and FT-IR spectrometer Thermo-Nicolet IR200. The mass spectra were measured with a CG-MS Shimadzu QP-2010 spectrometer with a HP-5 column. NMR spectra were measured at 298 K on a Bruker DPX 200 spectrometer. The compounds were dissolved in CDCl_3_. Chemical shifts, δ, are given in ppm relative to TMS (δ = 0 ppm) and are referenced by using the residual undeuterated solvent signal. Coupling constants, J, are reported in Hz, multiplicities being marked as: singlet (s), doublet (d), triplet (t), double triplet (dt) of multiplet (m). 

### 4.4. Toxins Isolation

The PLA_2_ from *C. d. cumanensis* was obtained from a pool venom of four specimens maintained in captivity at the serpentarium of the University of Antioquia (Medellín, Colombia).

The enzyme was purified through reverse-phase HPLC on C-18 column eluted at 1.0 mL/min with a gradient from 0% to 100% of acetonitrile in 0.1% trifluoroacetic acid (*v*/*v*). The absorbance in the effluent solution was recorded at wavelength of 280 nm [26].

Baxt-I was isolated from a venom pool collected from adult specimens of *B. atrox* from Meta, southeastern Colombia, via ion-exchange chromatography (CM-Sephadex C25) following the protocol described by Patiño et al. (2010) [27]. For both proteins, the purity was judged by RP-HPLC and SDS-PAGE.

### 4.5. Inhibition of PLA_2_ Activity Using 4-nitro-3-octanoyloxybenzoic acid (4N3OBA) as Monodispersed Substrate

The measurements of enzymatic activity using the monodispersed substrate 4N3OBA were performed according to the method described by Holzer and Mackessy (1996), and adapted for a 96-well ELISA plate. The standard assay contained 200 μL of buffer (10 mM Tris–HCl, 10 mM CaCl_2_, 100 mM NaCl, pH 8.0), 20 μL of 10 mM of substrate (4NO3BA), 20 μL of sample (20 μg PLA_2_ or 20 μg PLA_2_ + several concentrations of compounds) and 20 μL of water. The negative control was only buffer. The inhibitory effect of the studied thioesters on PLA_2_ activity was determined through co-incubation of the enzyme with each concentration of the compound for 30 min at 37 °C. After the incubation period, the sample was added to the assay and the reaction was monitored at 425 nm for 40 min (at 10 min intervals) at 37 °C. The quantity of chromophore released (4-nitro-3-hydroxy benzoic acid) was proportional to the enzymatic activity, and the IC_50_ value was determined from a logistic-dose response curve.

### 4.6. Inhibition of Proteolytic Activity

The inhibition of proteolytic activity was measured using the EnzCheck^®^ Gelatinase/Collagenase assay kit (Molecular Probes Inc., Eugene, Oregon, USA) following the protocol described by Preciado et al. (2017) with modification [28]. Briefly, aliquots of 80 μL of each triterpenic acid in concentrations from 15 to 500 μM, or buffer (0.05 M Tris-HCl, 0.15 M NaCl, 5 mM CaCl_2_, 0.2 mM sodium azide) as positive control, were added to each well of a 96-well plate. Then, 20 μL of DQ-gelatin followed by 100 μL of active Batx-I (1 μg/μL) were added, and the fluorescence intensity was measured by a Synergy HT Multi-Mode Microplate Reader (BioTek Instruments, Inc.; Winooski, USA) for excitation at 485 nm and emission detection at 515 nm at each minute for 60 min. Each reaction was performed in triplicate. 

### 4.7. Computational Studies

Compounds (I–III) were built using Gauss View 5 [29]. The geometric parameters for the more stable conformers were calculated at the B3LYP/6-31++G (d,p) level of approximation using GAUSSIAN 09 [30]. Molecular docking was carried out on a personal computer using Autodock Vina [31]. The structure of the PLA_2_ (PDB code 2QOG) from *Crotalus durissus terrificus* that showed 57% of homology with the PLA_2_ from *C. d. cumanensis* [24] was used in this study. 

Protein structure was prepared using the Protein Preparation module implemented in the Maestro program and uploaded without water molecules. Hydrogen atoms were automatically added to the protein according to the chemical nature of each amino acid, on the basis of the ionized form expected in physiological condition. This module also controls the atomic charges assignment. The 3D structure of the protein was relaxed through constrained local minimization, using the OPLS force fields in order to remove possible structural mismatches due to the automatic procedure employed to add the hydrogen atoms. When necessary, bonds, bond orders, hybridizations and hydrogen atoms were added, charges were assigned (a formal charge of +2 for Ca ion) and flexible torsions of ligands were detected.

To perform molecular docking experiments of compounds I–III with PLA_2_, the α-carbon of His48 was used as the center of the grid (X = 44.981, Y = 27.889 and Z = 46.392), whose size was 24 Å^3^. Exhaustiveness = 20. Finally, the ligand poses with best affinity were chosen, and a visual inspection of the interactions at the active site was performed and recorded using the open functionalities of Discovery Studio Visualizer and UCSF Chimera (www.cgl.ucsf.edu/chimera/). Physicochemical properties with importance in oral bioavailability for compounds I–III were calculated using Molinspiration [32]. 

### 4.8. Statistical Analysis

In order to determine significant differences between the concentrations of the compounds I–III used in the inhibition of the enzymes metalloproteinase and PLA_2_, two-way ANOVA followed by Bonferroni’s test was applied. In all cases, a difference with a *p* < 0.05 was considered significant.

## 5. Conclusions

Thioesters derived from 2-sulfenyl ethylacetate inhibited, in a specific way, PLA_2_ activity in micromolar concentrations. Although, we found that compounds II and III could inhibit the activity of Batx, IC_50_ values were around 10 times higher than those found for PLA_2_. It is suggested that inhibition of PLA_2_ activity is due to blockade of the active site and the interaction with amino acids involved in catalysis and binding substrate. Our results also suggest that compound III may be subject to further studies to develop inhibitors of snake venom PLA_2_. 

## Figures and Tables

**Table 1:** Studied compounds and physicochemical properties.

Compound	R3	R4	MW ^a^	nON ^a^	nOHNH ^a^	LogP(calc) ^a^
I	H	Cl	258.73	3	0	3.23
II	NO_2_	H	269.28	6	0	2.48
III	H	NO_2_	269.28	6	0	2.51

^a^ physicochemical properties calculated using Molinspiration. MW: molecular mass (Da). nON: hydrogen bonds acceptor. nOHNH: hydrogen bonds donator. LogP: calculated octanol/water partition coefficient.

**Table 2:** IC_50_ and confidence interval values for inhibition of phospholipases A_2_ (PLA_2_) and proteolytic activity.

Compound	IC_50_ Value for Inhibition of PLA_2_ Activity (μM)	95% Confidence Interval (μM)	IC_50_ Value for Inhibition of Proteolytic Activity (μM)	95% Confidence Interval (μM)
I	193.2 ^a^	133.5–279.5	-	-
II	305.4 ^b^	230.5–404.5	2774	2471–3115
III	132.7 ^a^	98.8–178.3	1879	1679–2102

^a^ No statistical differences between them (*p* > 0.05). ^b^ Statistical differences respect to other compounds (*p* < 0.05).

**Table 3:** Dihedral angle values for stable conformers.

Compound	0°		120°	
	δC-S-C=O	δC-O-C=O	δC-S-C=O	δC-O-C=O
I	5.0°	−0.8°	5.4°	−0.5°
II	4.7°	−0.5°	5.8°	0.0°
III	4.9°	−0.5°	5.8°	0.3°

Calculated with B3LYP/6-31++G(d,p) level of approximation.

## Supplementary Materials

NMR, IR and MS spectra of synthesized compounds are available as supplementary material, https://www.mdpi.com/1424-8247/12/2/80/s1.
