# Supplementary material for: Synthetic Inhibitors of Snake Venom Enzymes: Thioesters Derived from 2-Sulfenyl Ethylacetate
Source: Pharmaceuticals (Basel). 2019 May 23;12(2):80. doi: 10.3390/ph12020080 (PMC6630910; doi:10.3390/ph12020080)
Supplement: Supplementary file 1 [file pharmaceuticals-12-00080-s001.pdf]

pharmaceuticals

Article

Synthetic Inhibitors of Snake Venom Enzymes: Thioesters Derived From 2-Sulfenyl Ethylacetate

Isabel C. Henao Castañeda [1,*], Jaime A. Pereañez [2] and Lina M. Preciado [2]

SUPPLEMENTARY MATERIAL

Figure S1. ^{1}**H NMR Spectrum Ethyl 2-((4-chlorobenzoyl)thio)acetate (I)**

Figure S2. ^{13}**C NMR Spectrum Ethyl 2-((4-chlorobenzoyl)thio)acetate (I)**

Figure S3. ^{1}**H NMR Spectrum Ethyl 2-((3-nitrobenzoyl)thio)acetate (II)**

Figure S4. ^{13}**C NMR Spectrum Ethyl 2-((3-nitrobenzoyl)thio)acetate (II)**

Figure S5. ^{1}**H NMR Spectrum Ethyl 2-((4-nitrobenzoyl)thio)acetate (III)**

Figure S6. ^{13}**C NMR Spectrum Ethyl 2-((4-nitrobenzoyl)thio)acetate (III)**

Figure S7. GC-MS Ethyl 2-((4-chlorobenzoyl)thio)acetate (I)

Figure S8. GC-MS Ethyl 2-((3-nitrobenzoyl)thio)acetate (II)

Figure S9. GC-MS Ethyl 2-((4-nitrobenzoyl)thio)acetate (III)

Figure S10. IR Ethyl 2-((4-clorobenzoyl)thio)acetate (I)

Figure S11. IR Ethyl 2-((3-nitrobenzoyl)thio)acetate (II)

Figure S12. IR Ethyl 2-((4-nitrobenzoyl)thio)acetate (III)

Figure S1. ^{1}H NMR Spectrum Ethyl 2-((4-chlorobenzoyl)thio)acetate (I)

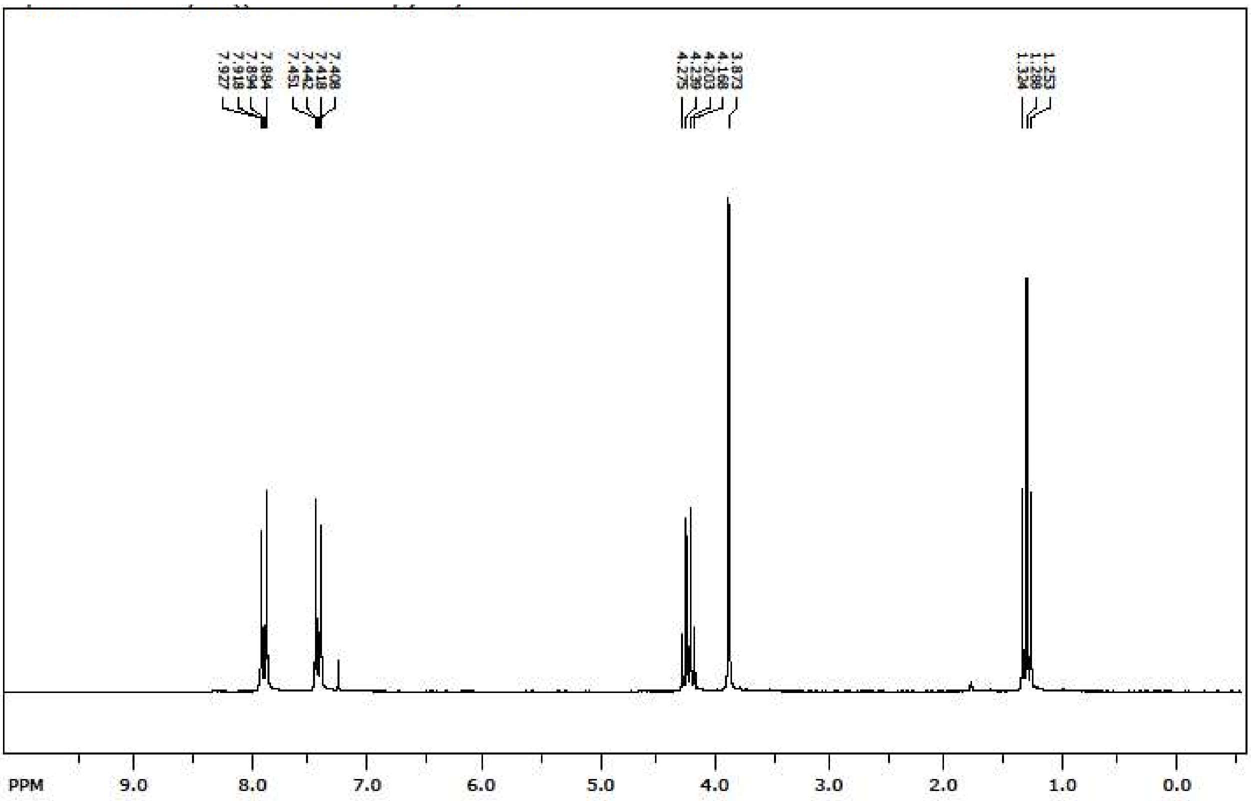

Figure S2. ^{13}C NMR Spectrum Ethyl 2-((4-chlorobenzoyl)thio)acetate (I)

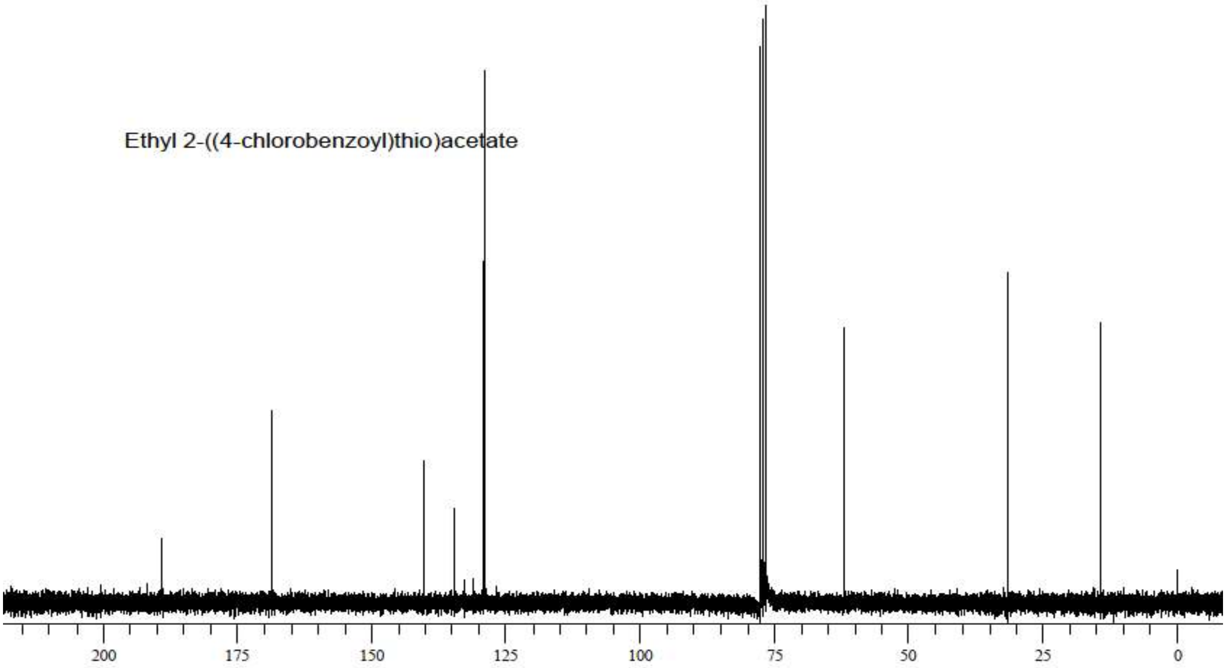

Figure S3. ^{1}**H NMR Spectrum Ethyl 2-((3-nitrobenzoyl)thio)acetate (II)**

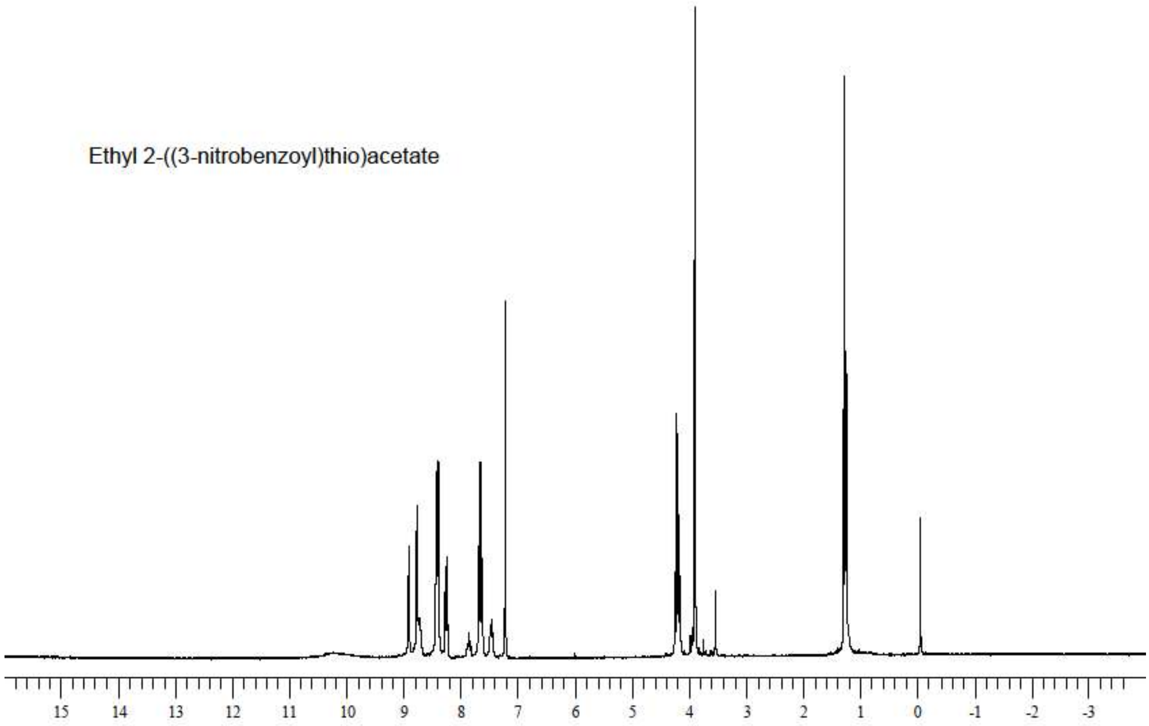

Figure S4. ^{13}**C NMR Spectrum Ethyl 2-((3-nitrobenzoyl)thio)acetate (II)**

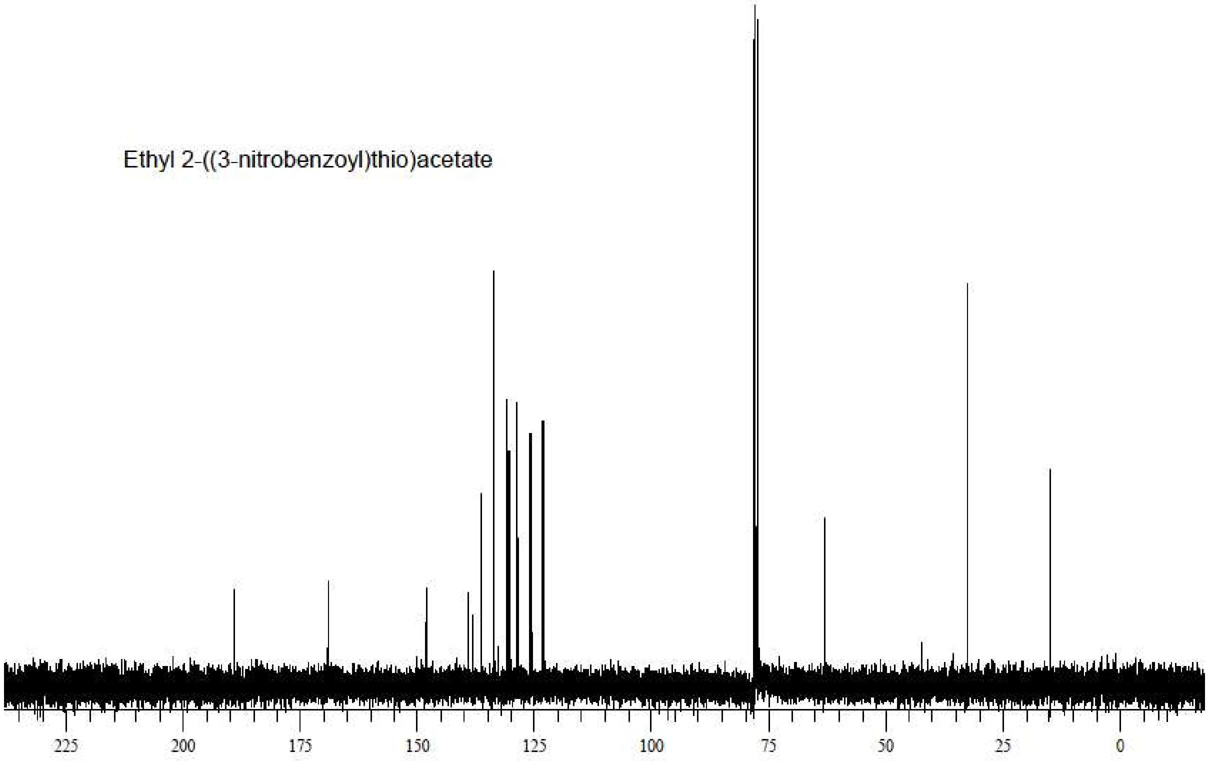

Figure S5. ^{1}H NMR Spectrum Ethyl 2-((4-nitrobenzoyl)thio)acetate (III)

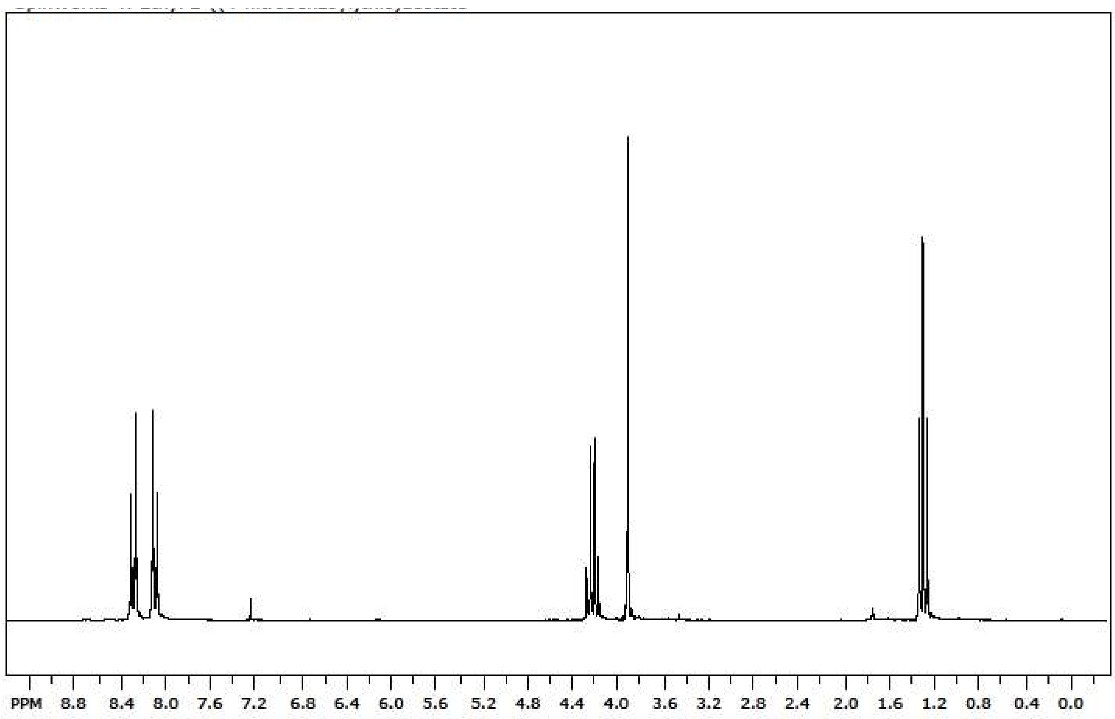

Figure S6. ^{13}C NMR Spectrum Ethyl 2-((4-nitrobenzoyl)thio)acetate (III)

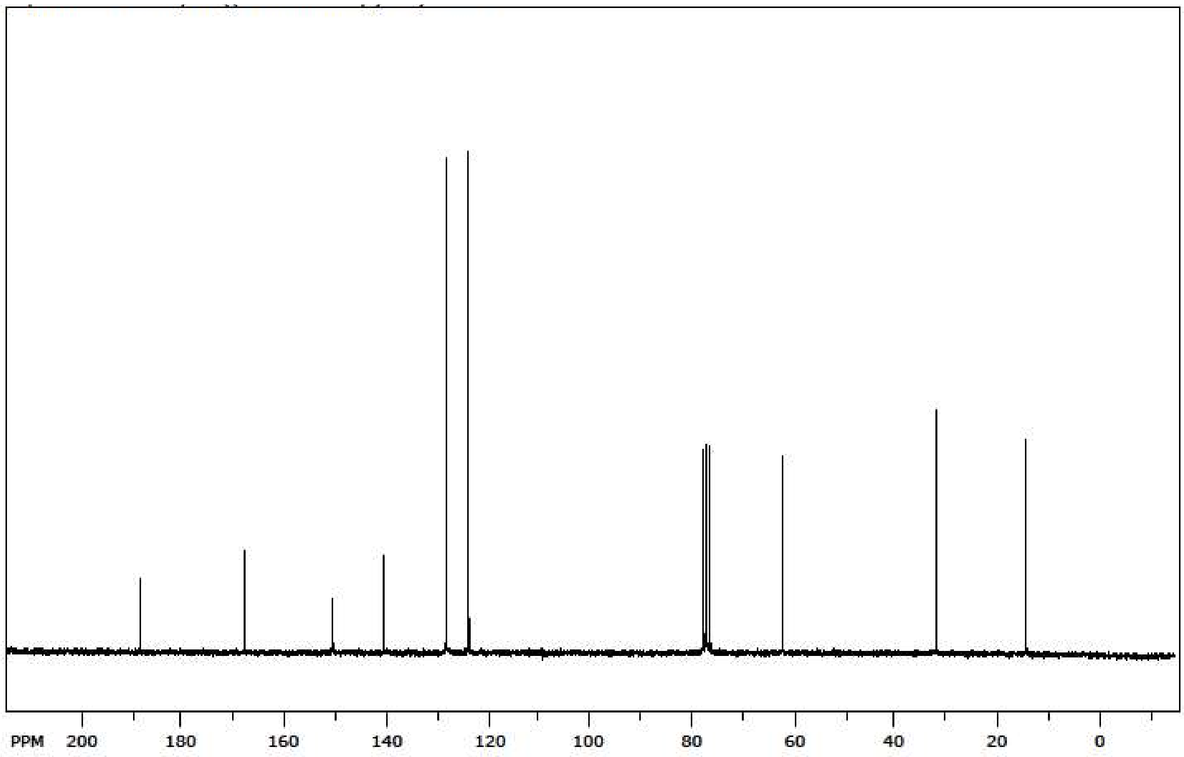

Figure S7. GC-MS Ethyl 2-((4-chlorobenzoyl)thio)acetate (I)

Ethyl 2-((4-chlorobenzoyl)thio)acetate

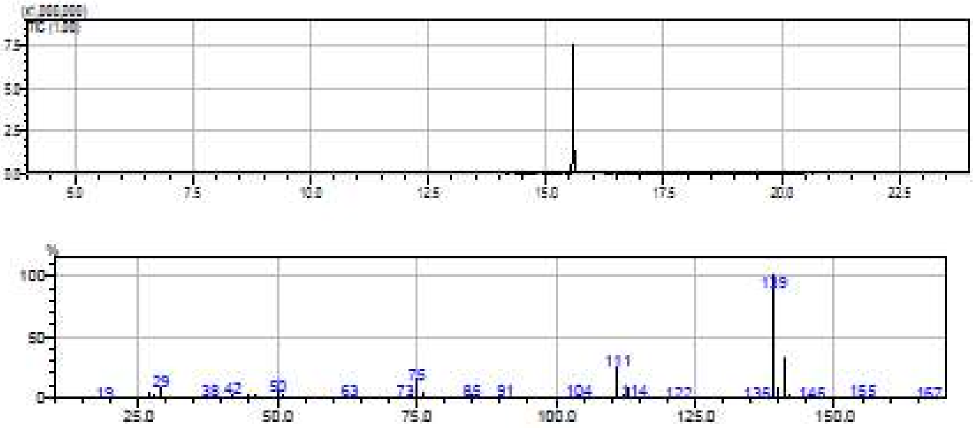

Method

[Comment]

===== Analytical Line 1 =====

[GC-2010]

Column Oven Temp.	:100.0 °C
Injection Temp.	:200.00 °C
Injection Mode	:Split
Flow Control Mode	:Pressure
Pressure	:100.0 kPa
Total Flow	:31.0 mL/min
Column Flow	:1.33 mL/min
Linear Velocity	:43.0 cm/sec
Purge Flow	:3.0 mL/min
Split Ratio	:20.0
High Pressure Injection	:OFF
Carrier Gas Saver	:OFF
Splitter Hold	:OFF

Oven Temp. Program

Rate	Temperature(°C)	Hold Time(min)
-	100.0	3.00
10.00	250.0	0.00
10.00	300.0	1.00

< Ready Check Heat Unit >
Column Oven : Yes
SPL1 : Yes
MS : Yes
< Ready Check Detector(FTD/BID) >
< Ready Check Baseline Drift >
< Ready Check Injection Flow >
SPL1 Carrier : Yes
SPL1 Purge : Yes
< Ready Check APC Flow >
< Ready Check Detector APC Flow >
External Wait :No
Equilibrium Time :0.0 min

[GC Program]

[GCMS-QP2010]

IonSourceTemp	:200.00 °C
Interface Temp.	:250.00 °C
Solvent Cut Time	:3.50 min
Detector Gain Mode	:Relative to the Tuning Result
Detector Gain	:1.08 kV +0.00 kV
Threshold	:50

[MS Table]
--Group 1 - Event 1--
Start Time :4.00min

Figure S8. GC-MS Ethyl 2-((3-nitrobenzoyl)thio)acetate (II)

Ethyl 2-((3-nitrobenzoyl)thio)acetate (II)

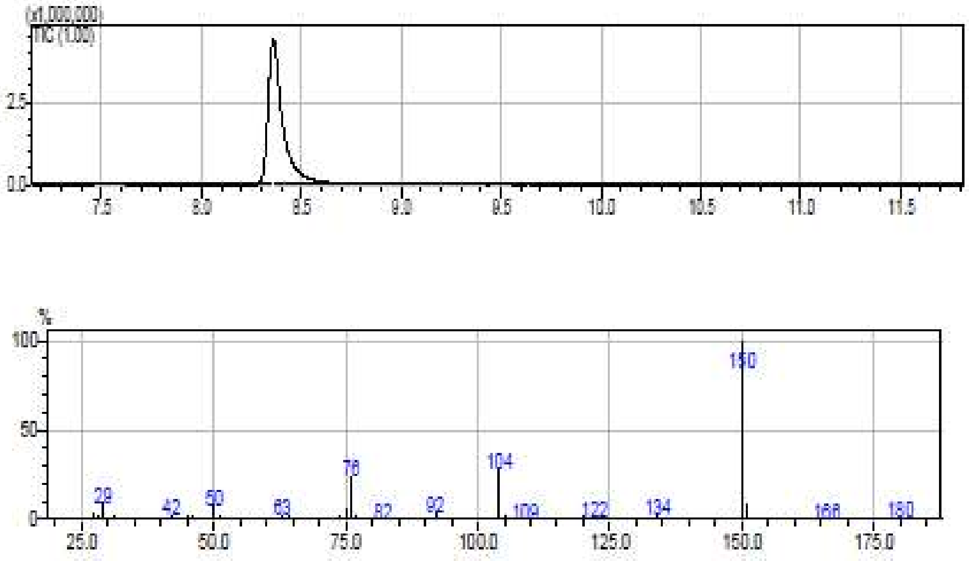

Method

[Comment]

——— Analytical Line 1 ———

[GC-2010]
Column Oven Temp. :200.0 °C
Injection Temp. :200.00 °C
Injection Mode :Split
Flow Control Mode :Pressure
Pressure :100.0 kPa
Total Flow :21.8 mL/min
Column Flow :0.90 mL/min
Linear Velocity :36.6 cm/sec
Purge Flow :3.0 mL/min
Split Ratio :20.0
High Pressure Injection :OFF
Carrier Gas Saver :OFF
Splitter Hold :OFF
Oven Temp. Program

Rate	Temperature(°C)	Hold Time(min)
-	200.0	2.00
5.00	300.0	2.00

< Ready Check Heat Unit >
Column Oven : Yes
SPL1 : Yes
MS : Yes
< Ready Check Detector(FTD/BID) >
< Ready Check Baseline Drift >
< Ready Check Injection Flow >
SPL1 Carrier : Yes
SPL1 Purge : Yes
< Ready Check APC Flow >
< Ready Check Detector APC Flow >
External Wait :No
Equilibrium Time :0.0 min

[GC Program]

[GCMS-QP2010]

Figure S9. GC-MS Ethyl 2-((4-nitrobenzoyl)thio)acetate (III)

Ethyl 2-((4-nitrobenzoyl)thio)acetate

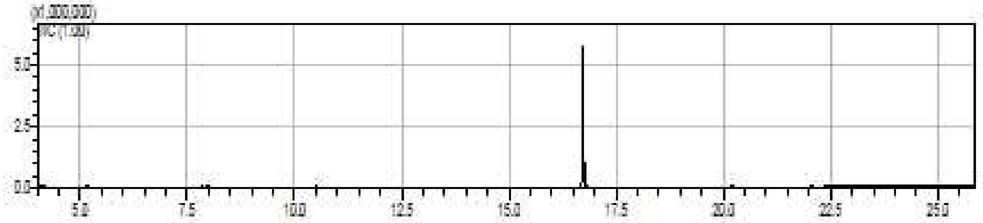

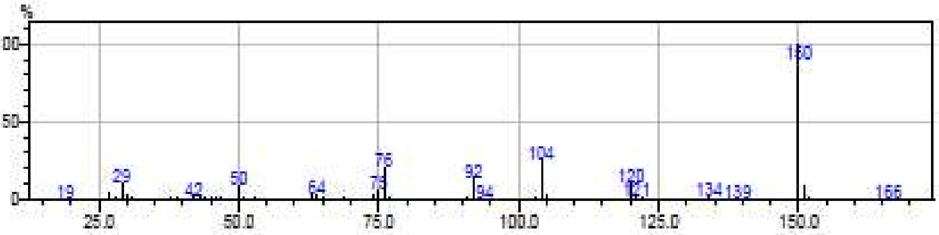

Method

[Comment]

===== Analytical Line 1 =====

[GC-2010]

Column Oven Temp.	:100.0 °C
Injection Temp.	:200.00 °C
Injection Mode	:Split
Flow Control Mode	:Pressure
Pressure	:100.0 kPa
Total Flow	:31.0 mL/min
Column Flow	:1.33 mL/min
Linear Velocity	:43.0 cm/sec
Purge Flow	:3.0 mL/min
Split Ratio	:20.0
High Pressure Injection	:OFF
Carrier Gas Saver	:OFF
Splitter Hold	:OFF

Oven Temp. Program

Rate	Temperature(°C)	Hold Time(min)
-	100.0	2.00
10.00	250.0	0.00
10.00	300.0	7.00

< Ready Check Heat Unit >
Column Oven : Yes
SPL1 : Yes
MS : Yes
< Ready Check Detector(FTD/BID) >
< Ready Check Baseline Drift >
< Ready Check Injection Flow >
SPL1 Carrier : Yes
SPL1 Purge : Yes
< Ready Check APC Flow >
< Ready Check Detector APC Flow >
External Wait :No
Equilibrium Time :0.0 min

[GC Program]

[GCMS-QP2010]

IonSourceTemp	:200.00 °C
Interface Temp.	:250.00 °C
Solvent Cut Time	:3.50 min
Detector Gain Mode	:Relative to the Tuning Result
Detector Gain	:1.08 kV +0.00 kV
Threshold	:50

[MS Table]

Figure S10. IR Ethyl 2-((4-clorobenzoyl)thio)acetate (I)

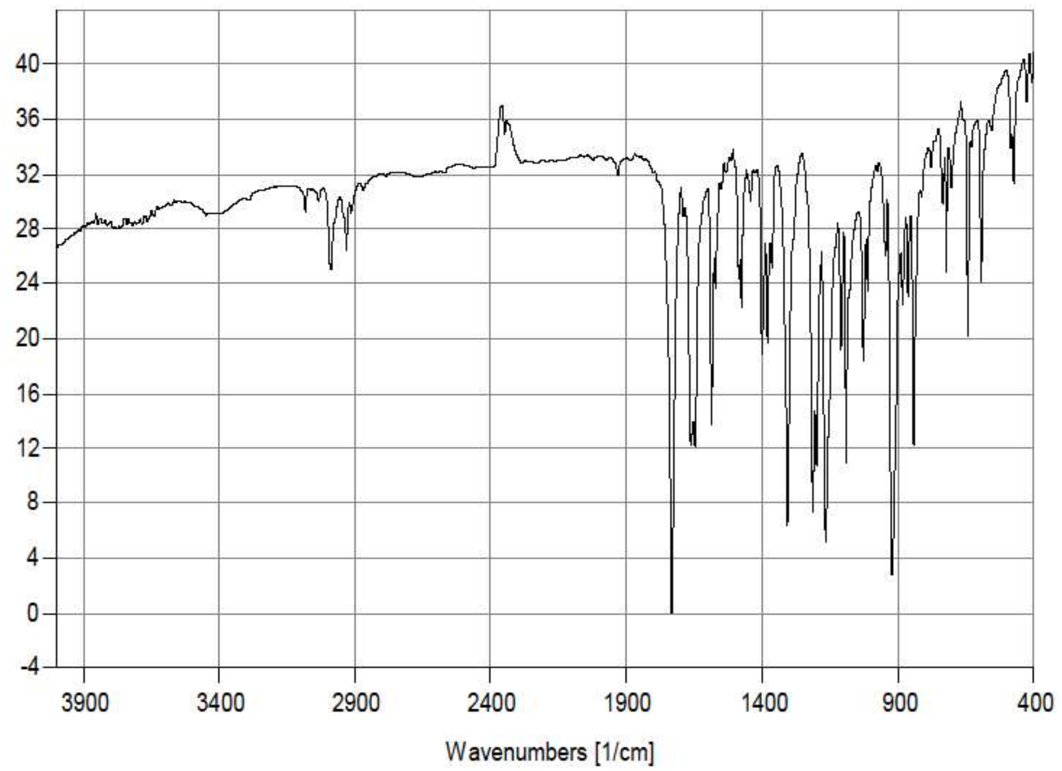

Figure S11. IR Ethyl 2-((3-nitrobenzoyl)thio)acetate (II)

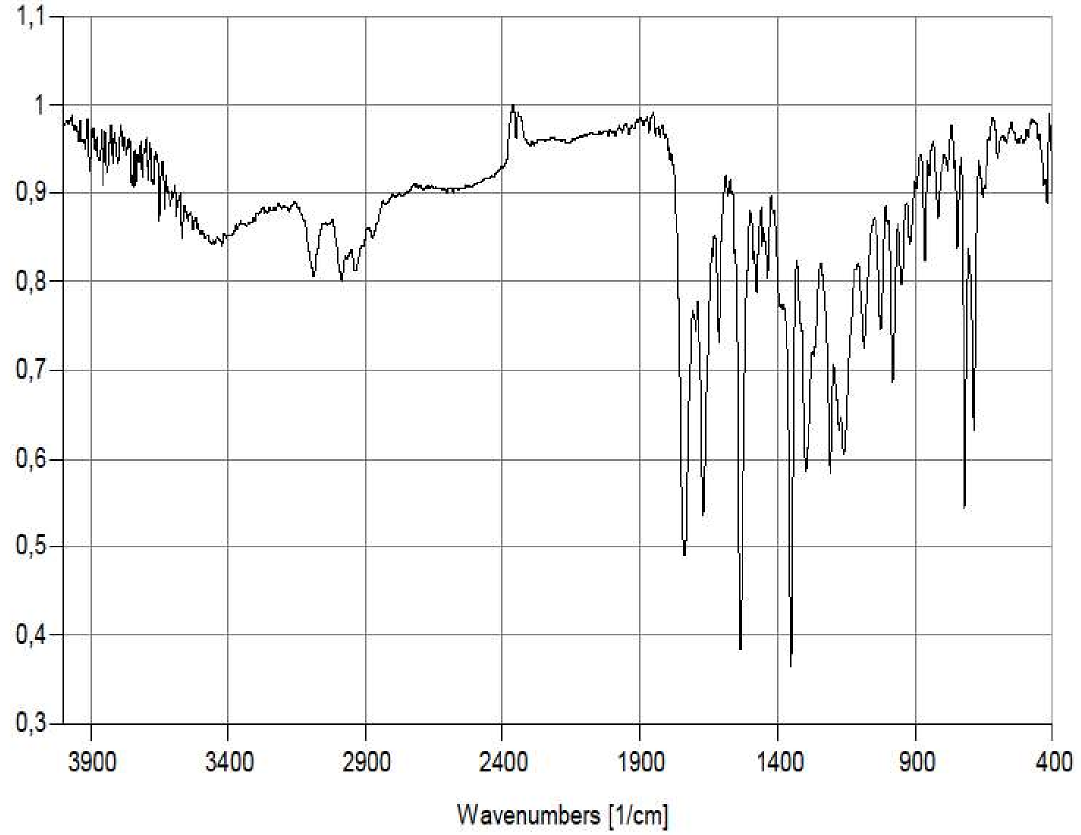

Figure S12. IR Ethyl 2-((4-nitrobenzoyl)thio)acetate (III)

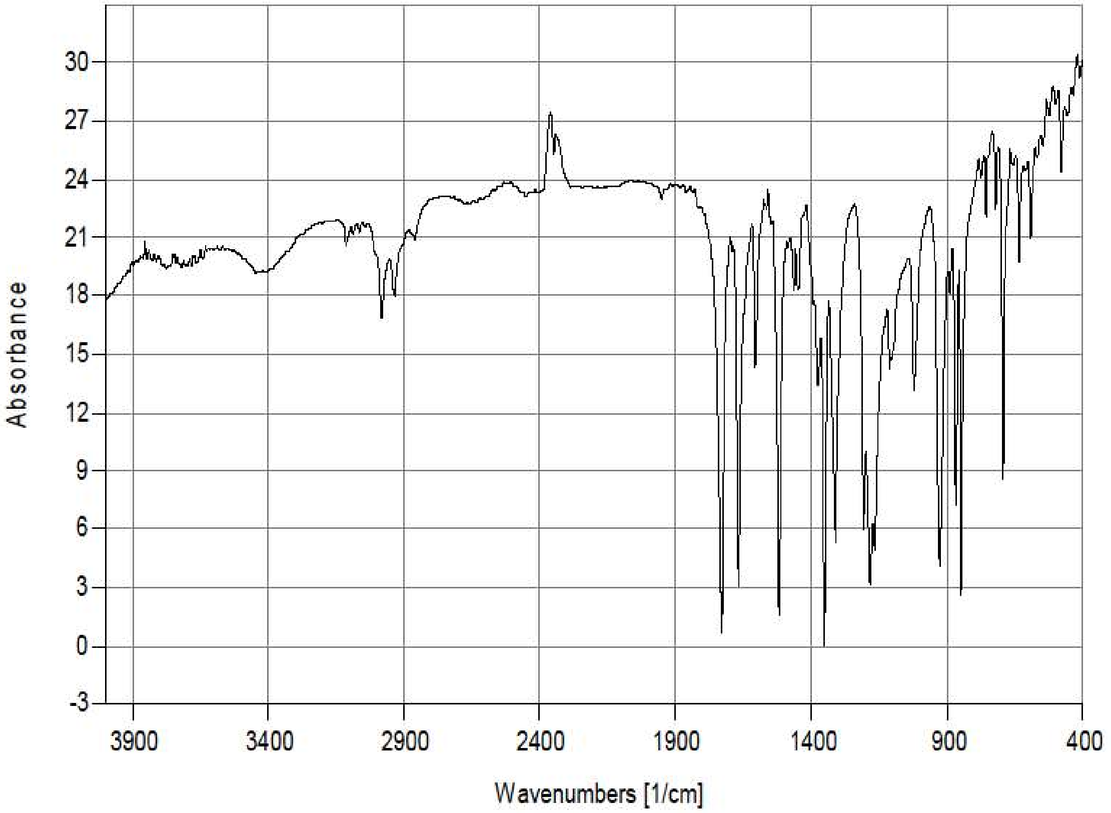

© 2019 by the authors. Licensee MDPI, Basel, Switzerland. This article is an open access article distributed under the terms and conditions of the Creative Commons Attribution (CC BY) license (http://creativecommons.org/licenses/by/4.0/).
